# Interaction of the tick immune system with transmitted pathogens

**DOI:** 10.3389/fcimb.2013.00026

**Published:** 2013-07-16

**Authors:** Ondřej Hajdušek, Radek Šíma, Nieves Ayllón, Marie Jalovecká, Jan Perner, José de la Fuente, Petr Kopáček

**Affiliations:** ^1^Biological Centre ASCR, Institute of ParasitologyČeské Budějovice, Czech Republic; ^2^SaBio. Instituto de Investigación en Recursos Cinegéticos IREC-CSIC-UCLM-JCCMCiudad Real, Spain; ^3^Faculty of Sciences, University of South BohemiaČeské Budějovice, Czech Republic; ^4^UMR INRA ONIRIS 1300 BioEpARNantes, France; ^5^Department of Veterinary Pathobiology, Center for Veterinary Health Sciences, Oklahoma State UniversityStillwater, OK, USA

**Keywords:** tick, tick-borne diseases, innate immunity, phagocytosis, antimicrobial peptides, *Borrelia*, *Anaplasma*, *Babesia*

## Abstract

Ticks are hematophagous arachnids transmitting a wide variety of pathogens including viruses, bacteria, and protozoans to their vertebrate hosts. The tick vector competence has to be intimately linked to the ability of transmitted pathogens to evade tick defense mechanisms encountered on their route through the tick body comprising midgut, hemolymph, salivary glands or ovaries. Tick innate immunity is, like in other invertebrates, based on an orchestrated action of humoral and cellular immune responses. The direct antimicrobial defense in ticks is accomplished by a variety of small molecules such as defensins, lysozymes or by tick-specific antimicrobial compounds such as microplusin/hebraein or 5.3-kDa family proteins. Phagocytosis of the invading microbes by tick hemocytes is likely mediated by the primordial complement-like system composed of thioester-containing proteins, fibrinogen-related lectins and convertase-like factors. Moreover, an important role in survival of the ingested microbes seems to be played by host proteins and redox balance maintenance in the tick midgut. Here, we summarize recent knowledge about the major components of tick immune system and focus on their interaction with the relevant tick-transmitted pathogens, represented by spirochetes (*Borrelia)*, rickettsiae (*Anaplasma)*, and protozoans (*Babesia)*. Availability of the tick genomic database and feasibility of functional genomics based on RNA interference greatly contribute to the understanding of molecular and cellular interplay at the tick-pathogen interface and may provide new targets for blocking the transmission of tick pathogens.

## Tick-pathogen interface: general considerations

Ticks are the most versatile arthropod diseases vectors capable to transmit the broadest spectrum of pathogens comprising viruses, bacteria, protozoa, fungi and nematodes to their vertebrate hosts (Jongejan and Uilenberg, 2004). The tick-borne diseases, such as Lyme disease, tick-borne encephalitis, rickettsiosis (spotted fever), ehrlichiosis or human granulocytic anaplasmosis, are of great concern in human health and their serious threat discourage people from outdoor work or leisure activities. No less important are tick-transmitted zoonoses, such as anaplasmosis, babesiosis, theileriosis and African swine fever that cause substantial economic losses to the livestock production worldwide.

The success rate of pathogens transmitted by ticks is mainly given by the favorable aspects of tick physiology arising from their adaptation to the relatively long-lasting blood feeding. The modulation of host immune and inflammatory responses by various bioactive molecules present in the tick saliva (Francischetti et al., 2009) facilitates pathogen acquisition and transmission. Furthermore, the long-term persistence of ingested microbes in the midgut lumen is facilitated by the absence of extracellular digestive enzymatic apparatus, which is in ticks located inside the digestive vesicles of midgut cells (Sonenshine, 1991; Sojka et al., 2013). Nevertheless, ticks possess defense mechanisms that allow them to maintain the pathogens and commensal microbes at the level, which does not impair their fitness and further development. The long lasting co-evolution of ticks with pathogens resulted in the mutual tolerance, apparently adapted to the tick physiological differences (Mans, 2011). Therefore, the detailed knowledge of tick physiology and behavior is crucial to understand the fate of pathogens within the tick vector. For instance, the length of feeding, that strikingly differs between the hard and soft ticks (days vs. minutes, respectively), definitely shape the course of pathogen transmission. Pathogens transmitted by the hard ticks (*Ixodidae*) usually undergo several days of development until they infect the host. On the contrary, pathogens transmitted by the soft ticks (*Argasidae*) are ready for transmission immediately after the feeding starts. A good example here is difference in the time of transmission between the *Borrelia* spirochetes causing Lyme disease (transmission several days after attachment) and relapsing fever (transmission several minutes after attachment) vectored by the hard and soft ticks, respectively (Sonenshine, 1991). Another important aspect that should be taken into consideration is the tick feeding strategy, the differences between one- and multi-host ticks in terms of transovarial and transstadial transmission.

**Figure 1:**
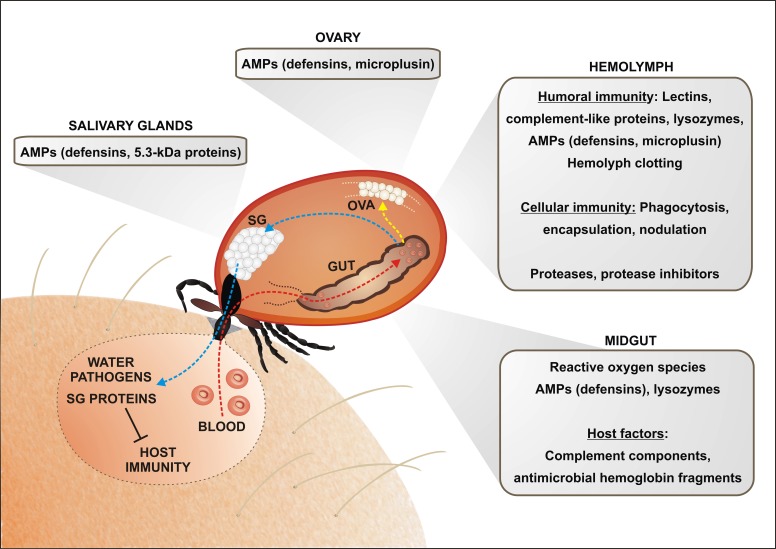
**An overview of the tick immune mechanisms and molecules constituting potential barriers for the pathogen transmission**. The pathogen transmission is tightly linked with physiology of blood feeding and tick innate immunity. Ingested blood meal is accumulated in the midgut content (red arrow; only one caecum shown). Hemoglobin and other proteins are taken up by the tick midgut cells and digested intracellularly in the lysosome-like digestive vesicles (Sojka et al., 2013). Liberated amino acids and other compounds are transported to the peripheral tissues and ovaries, supplying mainly egg development (yellow arrow). Importantly, the blood meal is concentrated by reabsorption of excessive water, which is spitted back into the wound by the action of salivary glands (blue arrow). Tick saliva contains a great variety of anti-coagulant, immunomodulatory and anti-inflammatory molecules that facilitate pathogen acquisition and transmission. The ingested pathogens have to survive the period between detachment and subsequent feeding of the next tick developmental stage and overcome several obstacles on its route through the tick body. In the midgut, tick may utilize some of the host immune molecules (e.g., complement system) for its own defense against intestinal inhabitants. Hemoglobin fragments, derived from the host hemoglobin, are secreted into the midgut lumen and exert strong antimicrobial activity. Tick midgut tissue also expresses a variety of endogenous AMPs, which sustain the midgut microbes at a tolerable level. An important, but still poorly understood, role is most likely played by the maintenance of the redox homeostasis in the tick midgut. Pathogens intruding into the tick hemocoel can be phagocytosed by tick hemocytes or destroyed by effector molecules of the humoral defense system, comprising AMPs, components of the primordial complement system (thioester-containing proteins (TEPs), convertase-like factors and fibrinogen-related lectins (FREPs). Ticks probably possess a mechanism of hemolymph clotting, but genes/proteins putatively involved in the activation of prophenoloxidase cascade leading to melanization have not yet been identified in any tick species. Tick salivary glands express also a variety of AMPs, which may impair pathogen acquisition and persistence in the tick, as demonstrated for the 5.3-kDa antimicrobial peptides and their role in the defense against *Anaplasma* infection (Liu et al., 2012). Abbreviations: GUT, midgut; OVA, Ovary; SG, salivary glands.

The transmitting pathogen acquired from the infected host has to overcome several tissue barriers within the tick body comprising midgut, hemocoel and salivary glands or ovary (in case of transovarial transmission). Each of these compartments may play a decisive role in the tick vector competence for a certain microbe. The tick midgut is probably the most important tissue for survival and proliferation of the pathogens since many of them have to persist here until the molting and subsequent feeding. On their route from the midgut to the peripheral tissues, the pathogens are facing cellular and humoral defense mechanisms functioning within the tick hemolymph. Therefore, the abilities to cope with or avoid the tick immune responses are crucial for the pathogen transmission. During the last two decades, our knowledge about the invertebrate immunity has rapidly expanded, mainly given by the research on the model organisms such as fruit fly *Drosophila melanogaster* (Ferrandon et al., 2007), horseshoe crab, crayfish or ascidians (Iwanaga and Lee, 2005; Söderhäll, 2010). A substantial progress has been also made in the field of blood feeders, such as mosquitoes (Osta et al., 2004; Hillyer, 2010) and tsetse flies (Lehane et al., 2004). The information on the tick innate immunity is rather fragmentary and allows only approximate comparison with other invertebrates (Sonenshine and Hynes, 2008; Kopacek et al., 2010). Nevertheless, even these scattered data indicate that ticks possess defense mechanisms protecting them against microbial infection (Figure 1). At the cellular level, they comprise phagocytosis, encapsulation and nodulation of foreign elements. The humoral defense is based on a variety of pattern-recognition proteins and effector molecules such as lectins, complement-related molecules and a broad spectrum of common as well as specific antimicrobial peptides (AMPs) (Kopacek et al., 2010). In addition, possibly important but rather unexplored role in the tick defense system is played by the immune molecules of the host origin.

In this review, we will follow the transmission routes of pathogens and subsequently enumerate the potential obstacles they have to evade in the tick body. The general features of tick immunity will be further discussed in relation to our current knowledge of tick interaction with the three most intensively studied agents of tick-borne diseases, represented here by *Borrelia* spirochete, intracellular rickettsia *Anaplasma*, and malaria-like protozoa *Babesia*.

## Tick immune system

### Tick midgut—the primary site of tick-pathogen interactions

Although the midgut of arthropod disease vectors is most likely the principle organ that determines their vector competence, the general knowledge of the mutual interplay between ingested pathogen, commensal microflora and tick itself is still inadequate. Unlike in mosquitoes and other insect blood feeders, the microbes ingested by ticks are not in direct contact with digestive proteases secreted into the lumen and the highly nutritious broth of concentrated blood proteins, neutral pH and long-term storage present an ideal environment for microbial proliferation. Therefore, ticks have to possess efficient defense mechanisms which maintain the intestinal microflora at tolerable level.

Two recent high-throughput mapping projects of the microflora (microbiome) by the next generation sequencing were carried out in two tick species, *R. microplus* (Andreotti et al., 2011) and *Ixodes ricinus* (Carpi et al., 2011). These studies revealed an extreme diversity of the bacterial community (more than hundred different organisms identified in one tick species), which apparently reflects tick geographical and environmental origin as well as developmental stage. However, encounter with a microbe, which a tick hardly meets in nature, could have a fatal consequence because of the lack of effective defense. A good example is the artificial infection of soft tick, *Ornithodoros moubata*, with the Gram (−) bacterium, *Chryseobacterium indologenes* (Buresova et al., 2006), which resulted in rapid tick death. Although this soft tick secretes into the midgut lumen at least two kinds of antimicrobial compounds protecting against Gram (+) bacteria, lysozyme (Kopacek et al., 1999; Grunclova et al., 2003) and defensins (Nakajima et al., 2001, 2002), these molecules apparently fail to protect the ticks against some Gram (−) bacteria. Defensins have been also frequently reported to be expressed in the midgut tissues of hard ticks (Hynes et al., 2005; Rudenko et al., 2005; Zhou et al., 2007), but their secretion and antimicrobial activity in the midgut lumen has not yet been unambiguously demonstrated. A defensin-related molecule named longicin, expressed in the midgut of *Haemaphysalis longicornis*, was reported to be active against a variety of microbes including Gram (+) and Gram (−) bacteria, fungi and various *Babesia* species (Tsuji et al., 2007) (see also below).

A specific role of the midgut defense against Gram (+) and some fungi is played by the antimicrobial activity of large peptides derived from the host hemoglobin (hemocidins). The antibacterial hemoglobin fragments were initially isolated from the midgut contents of the cattle tick *R. microplus* (Fogaca et al., 1999) and later also identified in the midgut of other soft and hard tick species (Nakajima et al., 2003; Sonenshine et al., 2005). The generation of antimicrobial hemoglobin fragments most likely occurs in the digestive cells during the initial phase of hemoglobin digestion by the synergic action of cathepsin D-type and cathepsin L-type aspartic and cysteine peptidases, respectively (Horn et al., 2009; Cruz et al., 2010).

Hemoglobin digestion and the concomitant process of heme detoxification via hemosome formation (Lara et al., 2003) is necessarily associated with the maintenance of the redox homeostasis in the tick midgut. Although this process is virtually unknown in ticks, the paradigm to follow is the recent seminal finding on the importance of redox balance in the mosquito midgut epithelial immunity. In the malaria vector *Anopheles gambiae*, a tandem of heme peroxidase and dual oxidase (Duox) catalyzes formation of dityrosine network between the midgut epithelium and lumen. This network prevents delivery of the epithelial immunity elicitors and ultimately results in up-regulation of intestinal microflora and *Plasmodium* infection in the lumen (Kumar et al., 2010). Heme peroxidase and NADPH oxidase 5 (Nox5) were further shown to mediate the epithelial nitration of *Plasmodium* ookinetes and hereby their opsonization for subsequent lysis by the complement-like action of thioester-containing protein TEP1 (Oliveira Gde et al., 2012). The redox situation may also indirectly affect the pathogen transmission by changing its balance with other microflora present in the midgut. An example, how the midgut microflora determines the competence of *A. gambiae* and malaria parasites was reported recently, showing that ROS produced by the mosquito midgut dweller *Enterobacter* sp. interfere with *Plasmodium* development (Cirimotich et al., 2011). The interrelationship between the redox balance and intestinal microflora could be quite complex, as demonstrated using sugar vs. blood fed mosquitoes *Aedes aegypti* (Oliveira et al., 2011). The presence of heme in the mosquito diet caused a significant decrease of ROS levels, resulting in consequent expansion of midgut bacteria. This phenomenon was interpreted as a result of the mosquito adaptation against the high oxidative stress potentially caused by reaction of pro-oxidative heme with high levels of continuously produced ROS (Oliveira et al., 2011).

By contrast, very little is known about the maintenance of redox homeostasis in the tick midgut except for one report showing the role of catalase in the regulation of the oxidative stress in the cattle tick *R. microplus* (Citelli et al., 2007) and the seminal work on the heme-detoxification pathway described in the same species (Lara et al., 2003, 2005). Nevertheless, the genomic and transcriptomics data from other tick species suggest that ticks do maintain the redox homeostasis in their midguts as they possess ROS-generating enzymes, such as NOX5 or DUOX, and arsenal of antioxidant enzymes and radical scavengers comprising catalases, glutathione- and thioredoxin peroxidases, glutathione S-transferases, and selenoproteins (Anderson et al., 2008; Megy et al., 2012). Thus, the framework of redox balance and its direct or indirect impact on the persistence of pathogens in the tick midgut offers almost unlimited inspiration for the further research.

## Immune reactions within the tick hemolymph

The major portion of our knowledge on the tick innate immunity is associated with cellular and humoral immune responses within the tick hemocoel. The volume of tick hemolymph increases linearly during the tick feeding from about 2–3 μl in unfed to almost 150 μl in fully engorged females, as demonstrated for *Dermacentor andersoni* (Kaufman and Phillips, 1973). At least three types of hemocytes, namely plasmatocytes, granulocytes I and granulocytes II, have been recognized in the hard and soft ticks, out of which the former two are phagocytic (Sonenshine, 1991; Borovickova and Hypsa, 2005). Several studies demonstrated the capability of hemocytes from different tick species to engulf foreign material and different microbes (Inoue et al., 2001; Loosova et al., 2001; Buresova et al., 2006). In addition, it was demonstrated that phagocytosis of microbes by the tick hemocytes is associated with humoral defense mechanisms, such as the production of ROS (Pereira et al., 2001) or complement-like molecules (Buresova et al., 2009, 2011). The process of hemocytic encapsulation of artificial implants, possibly linked with hemolymph coagulation and cellular response against *Escherichia coli* resembling nodulation, was reported to occur in the hemocoel of *Dermacentor variabilis* (Eggenberger et al., 1990; Ceraul et al., 2002).

Of special interest is the phagocytosis of tick-transmitted pathogens, such as *Borrelia* spirochetes, which seem to be engulfed at least in part by the process of “coiling” phagocytosis (Rittig et al., 1996). A comparison of the phagocytic and borreliacidal activity against *B. burgdorferi* injected into the hemocoel of natural vector, *I. scapularis* and a refractory tick *D. variabilis*, revealed much stronger immune response against the spirochetes in the latter immunocompetent tick species (Johns et al., 2000, 2001a). On the other hand, it was recently shown that infection of *I. scapularis* hemocytes by *A. phagocytophilum* is mediated by the protein named P11 and is required for successful migration of the pathogen from the midgut to salivary glands (Liu et al., 2011), meaning that phagocytosis or engulfment of the pathogen by tick hemocytes does not necessarily cause its elimination. This rise an interesting question whether at least some of the tick-transmitted pathogens may take an advantage of being engulfed by tick hemocytes to hide from the attack of humoral immune responses in the hemocoel.

Effector molecules of several types have been described in the tick hemolymph, out of which reports on tick defensins are the most frequent since they have been identified in a number of hard and soft tick species (Chrudimska et al., 2010; Kopacek et al., 2010). Moreover, the recent analysis of *I. scapularis* genome revealed an extensive expansion of genes encoding for defensins and defensin-like peptides divided into two multi-gene families referred to as scapularisins and scasins, respectively (Wang and Zhu, 2011). Typical mature defensins are ~4 kDa cationic peptides with a conserved pattern of six paired cysteins, derived by C-terminal cleavage after the furin (RVVR) motif from ~8 kDa pre-prodefensin. Tick defensins are usually active against Gram (+) bacteria and their interactions with transmitted pathogens [except for the above mentioned longicin (Tsuji et al., 2007)] have not been yet unequivocally demonstrated. Varisin, a defensin isolated from the hemolymph of *D. variabilis*, exerted a borreliacidal effect in combination with lysozyme (but not alone), which may in part explain the incompetence of this species to sustain *B. burgdorferi* spirochetes (Johns et al., 2001b). Interestingly, depletion of varisin from the *D. variabilis* hemolymph using RNA interference resulted in the significant reduction of *Anaplasma marginale* infection, indicating that the impact of defense mechanisms on a certain pathogen might be quite complex and not always predictable (Kocan et al., 2008b, 2009).

In addition to defensins, ticks possess a specific class of histidine- and cysteine-rich antimicrobial peptides of size about 10 kDa, namely hebraein identified in *Amblyoma hebraeum* (Lai et al., 2004) and microplusin isolated from the hemolymph of *R. microplus* (Fogaca et al., 2004). Unlike defensins, which kill bacteria in a detergent-like manner by disruption of bacterial membranes, the bacteriostatic effect of microplusin is based on its capacity to sequester copper required mainly for bacterial respiration (Silva et al., 2009). Another cysteine-rich antimicrobial peptide, unrelated to microplusin and referred to as ixodidin, was isolated from *R. microplus* hemocytes and its antibacterial activity was proposed to be linked to the inhibitory activity against serine proteases by yet unknown mechanism (Fogaca et al., 2006).

The process of self/nonself recognition within the tick hemolymph is believed to involve the interaction of tick lectins and carbohydrates associated with the invading microbes (PAMPs or pathogen-associated molecular patterns). The activity of lectins/hemagglutinins with preferential binding specificity for N-acetyl-D-hexosamines, sialic acids and glycoconjugates have been identified in the hemolymph of several hard and soft tick species (Grubhoffer et al., 2008; Sterba et al., 2011) and is mainly attributed to the presence of fibrinogen-related proteins (FREPs) related to Dorin M, isolated and characterized from the soft tick *O. moubata* (Kovar et al., 2000; Rego et al., 2006). In contrast to mammalian ficolins, Dorin M lacks the N-terminal collagen-like domain and is closely related to the lectins of tachylectin-5 type known to function as pattern recognition molecules in the horseshoe crab immune system (Gokudan et al., 1999; Kawabata and Tsuda, 2002; Ng et al., 2007). The genomes of *I. scapularis* and *I. ricinus* contain genes encoding for a variety of FREPs named Ixoderins that can be phylogenetically divided into three major groups (Rego et al., 2005; Kopacek et al., 2010). Although the role of FREP family members in the invertebrate immunity may be multifunctional, as recently suggested for gastropod mollusk (Hanington and Zhang, 2011), we hypothesize that at least some tick FREPs play a role in activation of tick complement system, components of which have been identified in ticks (Kopacek et al., 2012).

Ticks are unique among other invertebrates in that they possess representatives of all major classes of thioester-containing proteins (TEP) known in vertebrates and arthropods: (1) molecules related to α2-macroglobulins, (2) C3-components of complement system and (3) insect TEPs and (4) macroglobulin complement-related proteins (MCR) (Buresova et al., 2006). The pan protease inhibitors of α_2_-macroglobulin type were reported to be present in the hemolymph of soft and hard ticks (Kopacek et al., 2000; Saravanan et al., 2003; Buresova et al., 2009), where they presumably protect the ticks against undesired proteolytic attack of endogenous as well as exogenous proteases including those of invading microbes. The inhibition of metalloproteases secreted by the Gram (−) bacterium *C. indologenes* was shown to be functionally linked with phagocytosis of this bacteria by the tick hemocytes (Buresova et al., 2009). Further functional study of the tick TEPs suggested that phagocytosis of different bacteria by the tick hemocytes depends on non-redundant involvement of various tick TEPs with a central role of C3-like molecules (Buresova et al., 2011). Although nothing is known about interaction of the tick TEPs with tick-transmitted pathogens, the paradigm of *A. gambiae* TEP1 as a complement-like molecule, which determines the mosquito competence to *Plasmodium* parasites (Blandin et al., 2004, 2008), should stimulate further research in this area. In addition to the TEP family, genome of *I. scapularis* contains genes encoding for putative C3 convertases (Kopacek et al., 2012) having the multi-domain architecture similar to that of factor C2/Bf and LPS-sensitive Factor C activating the ancient complement-like system in the horseshoe crab (Zhu et al., 2005; Ariki et al., 2008). These preliminary results suggest that ticks possess features of a primitive complement system, which evolved on Earth at least one billion years ago (Nonaka and Kimura, 2006).

The existence of tick molecule related to the horseshoe crab Factor C, which primarily serves to trigger the limulus clotting cascade upon recognition of bacterial endotoxins (Kawabata, 2010), may suggest that ticks possess also a system for hemolymph coagulation. This suggestion was in part corroborated by the high throughput screening of immune-responsive genes in *D. variabilis* challenged with different bacteria. The most inducible immune gene found among others was transglutaminase, which acts as a crosslinking enzyme in the terminal phase of clotting mesh formation (Jaworski et al., 2010). However, with a possible exception of the previous observation, where a fibrous matrix was formed around the Epon-Araldite particles implanted under *D. variabilis* cuticle (Eggenberger et al., 1990), a convincing evidence of hemolymph clotting in ticks is still missing.

In contrast to other arthropods, ticks most likely lack the prophenoloxidase (PPO) activation system leading to melanization, because no PPO-related gene has been yet identified neither in the genome of *I. scapularis* (Megy et al., 2012) nor within the extensive EST datasets from other tick species (Kopacek et al., 2010).

## Immune reactions within the salivary glands

The tick salivary glands and components of tick saliva have been investigated foremost for their indispensable role in the modulation of host hemostasis, inflammation and immune response at the tick-host interface (Francischetti et al., 2009). The increasing number of salivary glands transcriptomes (sialomes) from the hard and soft ticks revealed the expression of a various AMPs, such as defensins, microplusin/hebraein and lysozymes, in this tissue (Mans et al., 2008; Karim et al., 2011). A defensin-like peptide named longicornisin was purified from the salivary glands of *H. longicornis* (Lu et al., 2010) and two different antimicrobial peptides unrelated to any known AMPs designated as Ixosin and Ixosin B were isolated from the salivary glands of *Ixodes sinensis* (Yu et al., 2006; Liu et al., 2008). However, it still has not been demonstrated whether these salivary glands AMPs are secreted into the tick saliva or hemolymph and if they directly interact with pathogens. The only exception is the 5.3-kDa antimicrobial protein, referred to as ISAMP and isolated from the saliva of *I. scapularis*, which exerts activity against Gram (−) and Gram (+) bacteria (Pichu et al., 2009). The transcripts encoding the family of secreted 5.3-kDa proteins were previously described to be significantly enriched in the transcriptome of *I. scapularis* nymphs infected with *B. burgdorferi* (Ribeiro et al., 2006). More recently, it was demonstrated that the 5.3-kDa family members were markedly upregulated in the salivary glands and hemocytes during *A. phagocytophilum* infection and were involved in the *I. scapularis* defense against this pathogen. Intriguingly, they were also shown to be effector molecules regulated by the JAK-STAT pathway (Liu et al., 2012) and although the *I. scapularis* genome contains also components of the putative Toll and Imd immune signaling pathways (Megy et al., 2012; Severo et al., 2013), the 5.3-kDa family regulation by JAK/STAT is the only so far described case of tick antimicrobial response controlled by a signaling pathway.

## RNA interference—an antiviral defense in ticks

The RNA interference (RNAi) is an ancient mechanism evolved for the inhibition of foreign genetic elements and precise regulation of the endogenous genes during organism development (Myers and Ferrell, 2005). The RNAi seems to work very well in the tick tissues (De La Fuente et al., 2007b) and the genome of *I. scapularis* contains all components important for the endogenous and exogenous RNAi machinery including dicers, argonauts, dsRNA binding proteins, exonucleases and surprisingly also RNA-dependent RNA polymerases (Kurscheid et al., 2009). The discovery that plant viruses encoded suppressors of the gene silencing machinery provided a strong support for RNAi function as a natural defense mechanism against viruses (Lindbo et al., 1993; Ratcliff et al., 1999). It was shown that viral proteins identified as suppressors in plants and insect cells were able to abrogate RNA silencing also in the tick cells (Garcia et al., 2006). In the context of tick immunity, we can speculate that RNAi could interfere directly with the viral infection or regulate production of antimicrobial peptides through the expression of microRNAs.

## Tick interactions with transmitted pathogens

### Borrelia

Lyme disease is an emerging human tick-borne disease of temperate climates with a concurrent distribution spanning North America and Eurasia. It is caused by *Borrelia* spirochetes related to *Treponema* and *Leptospira*, mainly by *Borrelia burgdorferi* sensu stricto in the US. and *B. burgdorferi* sensu stricto, *B. garinii*, and *B. afzelii* in Europe (Radolf and Samuels, 2010). Borreliosis in humans affects multiple body systems, producing a range of potential symptoms (Burgdorfer et al., 1989). The classical sign of early infection is circular, expanding, skin rash at the tick bite site called *erythema migrans*. Treatment with antibiotics is effective at this stage of infection. When left untreated, the spirochetes disseminate throughout the body and are associated with arthritis (*B. burgdorferi* sensu stricto), neurological symptoms (*B. garinii*) or dermatitis (*B. afzelii*) (Stanek et al., 2012). Although Lyme disease is intensively studied, an effective vaccine is still not available and annual incidence in many countries continues leading over other human vector-borne diseases (Bacon et al., 2008).

*Borrelia* spirochetes survive in an enzootic cycle involving three-host *Ixodes* ticks and small animals like rodents, birds and lizards (Steere et al., 2004). The spirochetes are usually not detected in larger mammals, which are essential in the tick life cycle as a source of sufficient amount of blood for feeding females (adult female of *Ixodes* ticks can take in total about one milliliter of blood Balashov, 1972), but complement system of the vertebrate innate immunity lyses most of the bacteria (De Taeye et al., 2013). Humans are not able to efficiently kill the pathogens and often get infected. However, they are mostly dead-end hosts for both the ticks and the pathogens. Transovarial transmission of *Borrelia* in ticks is not likely (Rollend et al., 2013) and people can contract Lyme disease only by feeding of infected nymphs or adults, where nymphs play a key role in the epidemiology of disease because of their small size and relatively short feeding time.

**Figure 2:**
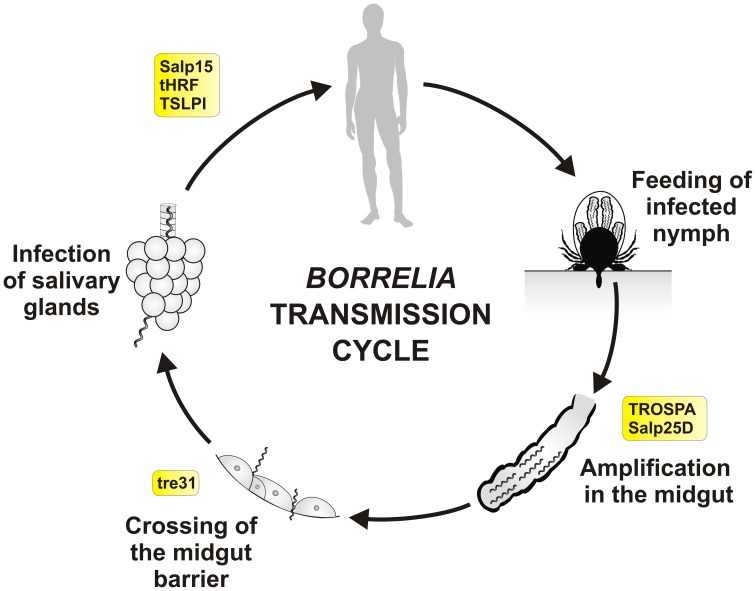
**Tick molecules involved in *Borrelia* transmission**. Schematic diagram representing general stages of *B. burgdorferi* sensu lato infection and transmission in the *Ixodes* nymph, the most important developmental stage for human infection. Tick molecules, for which interference with *Borrelia* acquisition, persistence and/or transmission have been proved by genetic tests, are shown in rectangles (see text and the Table 1 for their function and references). *Borrelia* spirochetes, that persisted in the tick midgut after the previous feeding (transstadial transmission), multiply rapidly within the newly engorged blood. They remain immobile and attached to the midgut cells. Around 53–72 h after the placement spirochetes become motile and swiftly cross the midgut barrier (between the cells), enter the hemolymph, salivary glands and via the saliva they infect new host (Ohnishi et al., 2001; Dunham-Ems et al., 2009). Transovarial transmission of *Borrelia* does not occur and newly hatched larvae are not infectious.

The interplay between tick proteins, *Borrelia* spirochetes and hosts has been mapped by transcriptomics and proteomics studies (Narasimhan et al., 2007a), antibody-screening assays (Das et al., 2001) and yeast-surface displays (Schuijt et al., 2011b). Several tick genes have been identified as crucial for acquisition of the infection in ticks, *Borrelia* persistence in the midgut and transmission into the next host during subsequent feeding (Figure 2 and Table 1). *Borrelia* colonization of the tick midgut lumen and their persistence until the next feeding is crucial process for the successful transmission of the parasite. *Borrelia* outer surface protein A (OspA) (De Silva et al., 1996), which is expressed predominantly inside the tick vector, is essential for pathogen adherence to the midgut cells during the acquisition phase and plays a significant role in the pathogen persistence. During the subsequent feeding, OspA expression is suppressed, but upregulated expression of OspC facilitates invasion of the tick salivary glands and transmission to the new host (Schwan et al., 1995). Tick protein called TROSPA (tick receptor for OspA) has been found to be implicated in the binding of OspA (Pal et al., 2004). TROSPA is specifically expressed in the midgut and its mRNA levels increase following the spirochete infection and decrease in response to engorgement. Importantly, interference with TROSPA expression by RNAi or its saturation by TROSPA antisera reduces *Borrelia* adherence to the midgut surface, preventing pathogen colonization of the vector and reducing its transmission (Pal et al., 2004).

**Table 1:** **Tick molecules interfering with *Borrelia* acquisition, persistence and/or transmission**.

**Name**	**Supposed function**	**RNAi effect on the pathogen**	**References**
Tick receptor for OspA (TROSPA)	Unknown	Reduced acquisition	Pal et al., 2004
Salivary protein 15 (Salp15)	Inhibition of the host complement system	Decreased transmission	Ramamoorthi et al., 2005
Salivary protein 25D (Salp25D)	Glutathione peroxidase	Decreased acquisition	Narasimhan et al., 2007b
Tick histamine release factor (tHFR)	Stimulation of histamine release	Decreased transmission	Dai et al., 2010
Tick receptor of BBE31 (tre31)	Unknown	Reduced persistence	Zhang et al., 2011
Tick salivary lectin pathway inhibitor (TSLPI, P8)	Inhibition of the host complement system	Decreased persistence and transmission	Schuijt et al., 2011a

An antibody-screening assay performed on rabbit sera with acquired resistance to the tick bites after *I. scapularis* infestation identified salivary gland protein called Salp25D (Das et al., 2001). Salp25D encodes for a glutathione peroxidase, is upregulated upon feeding, and silencing of this gene by RNAi or immunization of mice with the recombinant protein impairs spirochete acquisition by ticks (Narasimhan et al., 2007b). Thus, Salp25D is most likely important for quenching the reactive oxygen species released from the activated neutrophils and hereby protects *Borrelia* during acquisition and colonization of the tick midgut.

During the tick feeding, *Borrelia* spirochetes, which multiplied previously in the midgut content, cross midgut barrier (between the cells) to get into the hemolymph and salivary glands (De Silva and Fikrig, 1995; Hojgaard et al., 2008; Dunham-Ems et al., 2009). *Borrelia* enolase, an enzyme found on the surface of spirochetes, was shown to bind host plasminogen and facilitate dissemination of *Borrelia* in the ticks and host (Coleman et al., 1995, 1997). In the later study, yeast surface display approach identified that *Borrelia* outer-surface lipoprotein BBE31 interacted with the tick protein called tre31 (Zhang et al., 2011). Expression of tre31 is induced in the midgut upon *Borrelia* infection and silencing of tre31 by RNAi or blocking of BBE31 using mice antibodies decreases spirochete burden in the hemolymph and salivary glands of feeding ticks.

It has been shown before that proteins contained in the tick saliva had strong pharmacological properties, targeting coagulation, platelet aggregation, vasoconstriction (Chmelar et al., 2012) and complement system (De Taeye et al., 2013). Salp15 is a salivary gland protein with remarkable immunosuppressive properties, which is bound by *Borrelia* OspC surface protein during host invasion and protects the spirochetes from antibody-mediated killing (Ramamoorthi et al., 2005). Its expression is upregulated in salivary glands upon *Borrelia* infection and silencing of Salp15 by RNAi dramatically reduces the capacity of spirochetes to infect mice. Moreover, antibodies raised against tick Salp15 protect mice from the infection (Dai et al., 2009). Tick salivary lectin pathway inhibitor TSLPI, previously identified by yeast surface display assay as P8 protein with ability to reduce complement killing of *Borrelia* (Schuijt et al., 2011b), interferes with lectin complement pathway, resulting in impaired neutrophil phagocytosis and chemotaxis (Schuijt et al., 2011a). Silencing of this protein by RNAi or exposure of ticks to TSLPI-immunized mice decreases persistence of *Borrelia* in nymphs and hampers their transmission, respectively. Tick histamine-release factor tHRF is a saliva protein able to bind host basophils and stimulate histamine release (Dai et al., 2010). This property can be exploited by *Borrelia* spirochetes for host infection. Expression of tHRF is upregulated in *Borrelia*-infected ticks and silencing of this gene by RNAi or tHRF blocking by antibodies reduce tick feeding and decrease spirochete burden in mice. The last molecule that should be mention is tick salivary protein named Salp20, which is an inhibitor of alternative complement pathway and partially protects serum sensitive species of *Borrelia* from lysis (Tyson et al., 2007) by displacing properdin from C3 convertase (Tyson et al., 2008). However, functional genetic studies are needed to prove its role *in vivo*.

### Anaplasma

Anaplasmosis is considered as one of the most important vector-borne diseases of livestock (Kocan et al., 2010). The genus *Anaplasma* (Rickettsiales: Anaplasmataceae) includes six species of obligate intracellular bacteria, closely related to *Ehrlichia*, *Wohlbachia*, and *Neorickettsia*, occurring within the membrane-bound vacuoles called colonies in the host cytoplasm (Dumler et al., 2001; Kocan et al., 2008a). The *Anaplasma* rickettsiae preferably infect vertebrate red blood cells, however *A. phagocytophilum* attacks host neutrophils.

*A. phagocytophilum* infects a wide range of animals. It is responsible for the human granulocytic anaplasmosis (HGA), an emerging disease in the US, Europe and Asia, tick-borne fever in ruminants and equine and canine anaplasmosis (Woldehiwet, 2010). Three *Anaplasma* species exclusively infect ruminants: *A. marginale, A. centrale*, and *A. ovis*. *A. centrale* is used as life cattle vaccine in some regions, because infection with this parasite results only in mild clinical symptoms and could leave cattle persistently infected but immune against *A. marginale*, the causative agent of bovine anaplasmosis, which causes economic losses to the cattle industry worldwide. *A. ovis* is infective for sheep and wild ruminants, but infections are usually asymptomatic (Kocan et al., 2010). Also included in the genus *Anaplasma* are *A. bovis* and *A. platys*, which infect cattle and dogs, respectively.

All *Anaplasma* species are transmitted by Ixodid ticks, although tick transmissibility of *A. centrale* has been recently questioned (Shkap et al., 2009). The transmission cycle has been most extensively studied for *A. marginale* (Kocan et al., 1986, 1992a,b, 2008a). The developmental cycle in ticks is well coordinated with feeding and two *Anaplasma* morphotypes, reticulate (cell-dividing form) and dense core (infective form), can be found at each site of development (Kocan et al., 2010). Transovarial transmission of *Anaplasma* spp. from female ticks to their progeny does not occur. Therefore, ticks must acquire infection during blood feeding and the transmission cycles of these bacteria in nature are dependent upon the presence of infected reservoir hosts. Transmission by one-host ticks is probably accomplished by males, which can feed repeatedly and transfer between hosts (Sonenshine, 1991).

**Figure 3:**
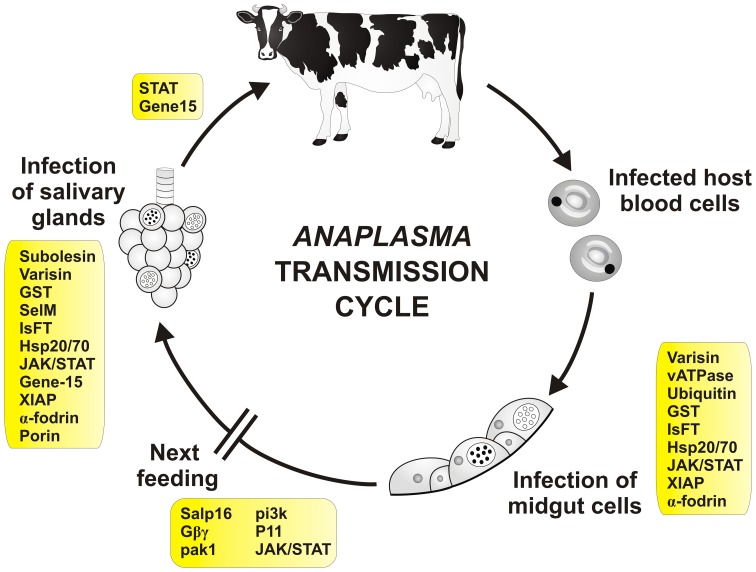
**Tick molecules involved in *Anaplasma* transmission**. Schematic diagram representing general stages of *Anaplasma* infection and transmission (Kocan et al., 2008a). Tick genes, for which interference with *Anaplasma* acquisition and/or transmission have been proved by genetic tests, are shown in rectangles (see text and the Table 2 for their function and references). Infected red blood cells (neutrophils for *A. phagocytophilum*) are engorged by the tick during blood meal. The released bacteria infect tick midgut cells and develop reticulate (cell-dividing; open circle) and dense core (infective; filled circle) forms of colonies inside the cells. During the next feeding, bacteria are released from the cells and infect other tissues including salivary glands. Here, they multiply inside the cells and are released into the saliva and transferred into the new host. Infection of tick hemocytes is required for the pathogen migration from the midgut to the salivary glands (Liu et al., 2011). Transovarial transmission of *Anaplasma* does not seem to occur. Transmission in one-host ticks is probably accompanied by tick males, which can feed repeatedly and transfer between hosts.

It has been shown that *Anaplasma* spp. modulate gene expression in ticks (De La Fuente et al., 2007a; Kocan et al., 2008a; Zivkovic et al., 2009; Sultana et al., 2010; Villar et al., 2010a,b), although differences may exist between species (Zivkovic et al., 2009). Functional studies of tick-*Anaplasma* interactions have shown how tick genes may affect bacterial infection (Figure 3 and Table 2). Four differentially regulated genes/proteins, glutathione S-transferase (GST), salivary selenoprotein M (SelM), vATPase, and ubiquitin have been identified by suppression-subtractive hybridization and differential in-gel electrophoresis analyses using tick IDE8 cells infected with *A. marginale* (De La Fuente et al., 2007a). Glutathione S-transferases are intracellular enzymes with various functions, mostly accompanying cellular detoxification, but also signaling (Oakley, 2011). Selenoproteins are selenocysteine-containing proteins and important antioxidants (Reeves and Hoffmann, 2009). Vacuolar H^+^ ATPases are membrane proteins acidifying a wide array of intracellular organelles by pumping protons across the plasma membranes (Nelson, 2003). Finally, ubiquitins are small regulatory proteins, involved in an intracellular destruction and recycling of proteins in the proteasome, which is an important process also for the regulation of arthropod immune pathways (Ferrandon et al., 2007). Silencing of GST, vATPase or ubiquitin by RNAi decreases midgut *Anaplasma* acquisition in *D. variabilis* males fed on *A. marginale* infected cows, while silencing of GST or SelM decreases pathogen infection in the salivary glands of infected ticks fed on naïve sheep (De La Fuente et al., 2007a; Kocan et al., 2009). As previously mentioned, silencing of *D. variabilis* defensin named varisin that was shown to be expressed primarily in hemocytes, but also in midgut and other tissues (Hynes et al., 2008), decreased midgut pathogen acquisition in *D. variabilis* males fed on *A. marginale* infected cows and decreased infection in salivary glands of infected ticks fed on naïve sheep with obvious morphological abnormalities in bacterial colonies (Kocan et al., 2008b). Moreover, silencing of E3 ubiquitin ligase named x-linked inhibitor of apoptosis (XIAP) increases colonization of *I. scapularis* midgut cells and salivary glands by *A. phagocytophilum*, attracting even more attention to the ubiquitination process in ticks (Severo et al., 2013).

**Table 2:** **Tick molecules interfering with *Anaplasma* acquisition and/or transmission**.

**Name**	**Supposed function**	**RNAi effect on the pathogen**	**References**
Salivary protein 16 (Salp16)	Unknown	Decreased acquisition	Sukumaran et al., 2006
Subolesin (SUB)	Component of the immune signaling pathways	Decreased acquisition	De La Fuente et al., 2006, 2008; Merino et al., 2011; Busby et al., 2012
Varisin	Defensin	Decreased acquisition	Kocan et al., 2008b
Vacuolar H^+^ ATPase (vATPase)	Acidification of vesicles	Decreased acquisition	De La Fuente et al., 2007a; Kocan et al., 2009
Ubiquitin	Protein degradation	Decreased acquisition	De La Fuente et al., 2007a; Kocan et al., 2009
Glutathione S-transferase (GST)	Detoxification and signaling	Decreased acquisition	De La Fuente et al., 2007a; Kocan et al., 2009
Salivary selenoprotein M (SelM)	Protection against oxidative stress	Decreased acquisition	De La Fuente et al., 2007a; Kocan et al., 2009
α-1,3 fucosyltransferase (IsFT)	Glycosylation of proteins	Decreased acquisition	Pedra et al., 2010
G protein-coupled receptor Gβγ subunits (Gβγ)	Signal transduction	Decreased acquisition	Sultana et al., 2010
Phosphoinositide 3-kinase (pak1)	Cytoskeletal reorganization and signaling	Decreased acquisition	Sultana et al., 2010
p21-activated kinase (pi3k)	Cytoskeletal reorganization and signaling	Decreased acquisition	Sultana et al., 2010
Protein 11 (P11)	Unknown	Decreased acquisition	Liu et al., 2011
Heat-shock protein 20 (Hsp20)	Cellular stress response	Increased acquisition	Busby et al., 2012
Heat-shock protein 70 (Hsp70)	Cellular stress response	Decreased acquisition	Busby et al., 2012
Janus kinase (JAK)	Component of JAK/STAT signaling pathway	Increased acquisition	Liu et al., 2012
Signal transducer and activator of transcription (STAT)	Component of JAK/STAT signaling pathway	Increased acquisition and transmission	Liu et al., 2012
Gene-15	Antimicrobial peptide	Increased acquisition and transmission	Liu et al., 2012
X-linked inhibitor of apoptosis protein (XIAP)	E3 ubiquitin ligase	Increased acquisition	Severo et al., 2013
α-fodrin (CG8)	Spectrin α-chain	Decreased acquisition	Ayllón et al., 2013
Porin (T2)	Mitochondrial voltage-dependent anion-selective channel	Decreased acquisition	Ayllón et al., 2013

Fucosylation, which participates in many pathological processes in eukaryotes, has been shown to be modulated in ticks during *Anaplasma* infection (Pedra et al., 2010). *A. phagocytophilum* modulates expression of *I. scapularis* α-1,3 fucosyltransferase (IsFT) and uses α-1,3-fucosylation process to colonize the tick vector. Silencing of IsFT by RNAi reduces acquisition but not transmission of *A. phagocytophilum* in ticks.

The arthropod immune responses are generally regulated by Toll, Imd and JAK/STAT pathways (Ferrandon et al., 2007). Janus kinase (JAK)/signaling transducer activator of transcription (STAT) pathway has been shown to play a critical role in the tick defense against *Anaplasma* (Liu et al., 2012). Silencing of JAK/STAT genes by RNAi in *I. scapularis* causes burden of *A. phagocytophilum* in midgut, hemolymph and SG. The gene-15 of the salivary glands family encoding a member of 5.3-kDa antimicrobial peptide family is highly induced upon *Anaplasma* infection and regulated by JAK/STAT pathway. Silencing of gene-15 (and also STAT) by RNAi causes increased infection in salivary glands and transmission to the mammalian host.

Salivary protein 16 (Salp16) is an antigen recognized by tick-exposed host sera. Silencing of Salp16 by RNAi does not influence *A. phagocytophilum* acquisition in *I. scapularis* midgut, but the pathogen is not able to successfully infect the salivary glands (Sukumaran et al., 2006). Furthermore, expression of Salp16 in the tick salivary glands is upregulated upon *Anaplasma* infection. It has been elegantly shown that Salp16 upregulation is not part of the tick defense mechanisms, but that *Anaplasma* selectively alter Salp16 expression for its benefit (Sultana et al., 2010). *A. phagocytophilum* infection induces actin phosphorylation, which is dependent on tick p21-activated kinase (ipak1)-mediated signaling. Activity of ipak1 is stimulated via G protein-coupled Gβγ receptor subunits (Gβγ), which in turn mediate phosphoinositide 3-kinase (pi3k) activation. In association with RNA polymerase II (RNAPII) and TATA box-binding protein, expression of Salp16 is selectively promoted. Silencing of ipak1, Gβγ or pi3k by RNAi reduces actin phosphorylation and *Anaplasma* acquisition by ticks (Sultana et al., 2010).

Recently, α-fodrin (spectrin α-chain) and mitochondrial porin (voltage-dependent anion-selective channel) were shown to be involved in *A. phagocytophilum* infection/multiplication and the tick cell response to infection in *I. scapularis* (Ayllón et al., 2013). The pathogen presence decreases expression of α-fodrin in the tick salivary glands and porin in both the midgut and salivary glands to inhibit apoptosis, subvert host cell defenses and increase infection. In the midgut, α-fodrin upregulation was used by the pathogen to increase infection due to cytoskeleton rearrangement that is required for pathogen infection. These results demonstrated that the pathogen uses similar strategies to establish infection in both vertebrate and invertebrate hosts.

After the initial infection of midgut cells, *Anaplasma* spread to other tick organs. However, the exact mechanism mediating migration to and infection of different tick organs is still not well known. Secreted *I. scapularis* protein 11 (P11), induced upon *A. phagocytophilum* infection, was shown to be important for *Anaplasma* migration from the midgut to the salivary glands, while being engulfed and hidden in the tick hemocytes (Liu et al., 2011). Silencing of P11 by RNAi or blocking the P11 with anti-sera or inhibition of hemocyte phagocytosis by injection of polystyrene beads into the tick hemolymph resulted in decreased *Anaplasma* infection of the tick salivary glands (Liu et al., 2011).

Tick subolesin (SUB), an ortholog of insect and vertebrate akirins, is possibly involved in several pathways, including innate immune responses, through a regulatory network involving cross-regulation between NF-κ B (Relish) and SUB and SUB auto-regulation (Naranjo et al., 2013). SUB is down-regulated during *A. phagocytophilum* infection of tick nymphs, but up-regulated in female midguts and salivary glands infected with *A. marginale* or *A. phagocytophilum* (De La Fuente et al., 2006; Galindo et al., 2009; Zivkovic et al., 2010; Merino et al., 2011; Busby et al., 2012). Silencing of SUB by RNAi has strong effect on tick mortality and feeding and causes degeneration of midgut, salivary glands and reproductive organs (De La Fuente et al., 2008). After SUB knockdown, infection with *A. marginale* is significantly reduced in *D. variabilis* male salivary glands, but has only little effect on infection with *A. phagocytophilum* (De La Fuente et al., 2006; Ayllón et al., 2013). Subolesin has been used for vaccination against tick infestations and pathogen infection (De La Fuente et al., 2011). Although limited success has been obtained in this area, ongoing efforts are focused on the characterization of the *Anaplasma*-tick interface to develop vaccines for the control of tick infestations and pathogen transmission (De La Fuente, 2012).

### Babesia

Babesiosis is a tick-borne malaria-like disease affecting health of many animals and reducing cattle production in tropical and subtropical regions worldwide. Moreover, human babesiosis increasingly raises public health concern (Florin-Christensen and Schnittger, 2009). *Babesia*, the causative agent of babesiosis, is an apicomplexan parasite, which is together with *Theileria* referred to as piroplasm because of its pear-shape intra-erythrocytic stage. The genus *Babesia* constitutes paraphyletic group of parasites (described in various hosts with discrepancies in developmental cycles), which can be only distinguished by an appropriate molecular methods (Allsopp and Allsopp, 2006). They multiply in vertebrate erythrocytes (asexual stage) and cause severe symptoms related to their destruction. In the tick (hard tick), the parasite undergo sexual development in the midgut content, multiply in midgut cells and spread to different tissues including the salivary glands and ovary. Most of *Babesia*, unlike *Theileria*, are capable of transovarial transmission and newly hatched larvae are infectious to the hosts (Chauvin et al., 2009; Florin-Christensen and Schnittger, 2009).

**Figure 4:**
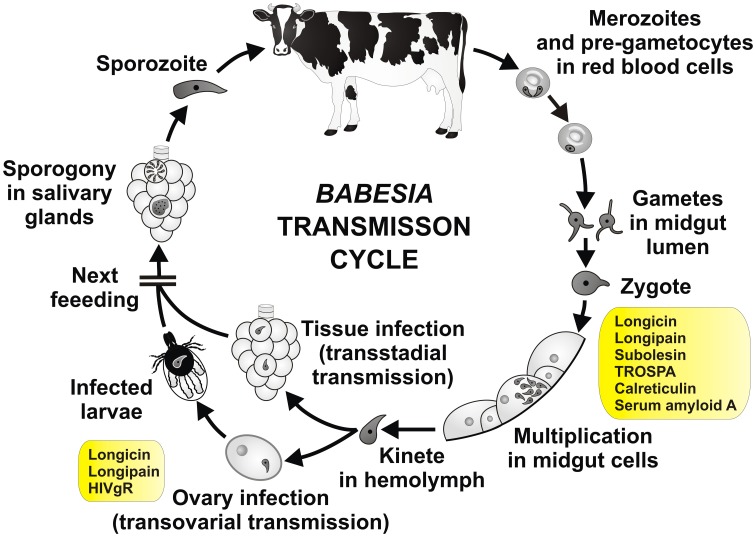
**Tick molecules involved in *Babesia* transmission**. Schematic diagram representing general stages of *Babesia* infection and transmission (Zintl et al., 2003; Chauvin et al., 2009; Florin-Christensen and Schnittger, 2009). Tick genes, for which interference with *Babesia* acquisition and/or transmission have been proved by genetic tests, are shown in rectangles (see text and the Table 3 for their function and references). Pre-gametocytes in red blood cells, taken up within the blood meal, develop in the tick midgut content into matured gametocytes and gametes (ray bodies, Strahlenkörper) with distinctive spine-like projections. They fuse and give raise to the spherical spiked zygotes, which invade midgut cells. Inside the midgut cells, the zygotes transform, undergo meiosis and differentiate into motile prolonged kinetes (ookinetes). The kinetes escape the midgut cells, enter the hemolymph and invade other tick tissues, including ovary (transstadial and transovarial transmission, respectively). Here they undergo asexual reproduction and produce sporokinetes, which further spread the infection inside the tick or newly emerged larvae. During the next feeding, the kinetes that invaded salivary glands undergo a final cycle of multiplication (sporogony) to produce numerous sporozoites, host-invasive stages of the parasite. The sporozoites enter the tick saliva and infect host.

It has been shown that infection of ticks with *Babesia* parasite pose negative effect on the tick development (Cen-Aguilar et al., 1998), thus ticks are supposed to evolve defense mechanisms to control *Babesia* infection and to regulate their mutual interaction. Although genomic sequences of *Babesia* and tick are available (Pagel Van Zee et al., 2007; Cornillot et al., 2012) and several projects have identified tick genes differently expressed upon *Babesia* infection (Rachinsky et al., 2007, 2008; Antunes et al., 2012; Heekin et al., 2012), only few tick genes have been shown to be directly implicated in the vector-pathogen interaction (Figure 4 and Table 3). First of them, called longicin (Tsuji and Fujisaki, 2007), is defensin-like protein of *H. longicornis* exerting anti-microbial and anti-fungal activity. Recombinant longicin was reported to inhibit proliferation of *Babesia* (*Theileria*) *equi* merozoites in *in vitro* cultures and to reduce parasitemia of mice experimentally infected with *B. microti*. Moreover, silencing of this gene by RNAi increased number of *B. gibsoni* in the tick midgut content, ovary and laid eggs, pointing to longicin role in the regulation of *H. longicornis* vectorial capacity (Tsuji et al., 2007).

**Table 3:** **Tick molecules interfering with *Babesia* acquisition and/or transmission**.

**Name**	**Supposed function**	**RNAi effect on the pathogen**	**References**
Longicin	Defensin	Increased acquisition and transovarial transmission	Tsuji et al., 2007
Longipain	Cysteine protease	Increased acquisition and transovarial transmission	Tsuji et al., 2008
*H. longicornis* vitellogenin receptor (HIVgR)	Uptake of vitellogenin	Decreased transovarial transmission	Boldbaatar et al., 2008
Subolesin (SUB)	Component of the immune signaling pathways	Decreased acquisition	Merino et al., 2011
Tick receptor for OspA (TROSPA)	Unknown	Decreased acquisition	Antunes et al., 2012
Calreticulin	Protein folding and signaling	Decreased acquisition	Antunes et al., 2012
Serum amyloid A	Response to inflammation	Decreased acquisition	Antunes et al., 2012

Longipain (Tsuji et al., 2008) is midgut-specific cysteine protease of *H. longicornis*, whose expression is upregulated upon blood feeding. Similarly as for longicin, recombinant protein inhibited proliferation of *Babesia* (*Theileria*) *equi* merozoites in *in-vitro* cultures and silencing of this gene by RNAi resulted in increased number of parasites in the midgut lumen, ovary, and hatched larvae. In general, inhibition of tick and parasite proteases is of interest as both the tick and the parasite genomes encode for several cysteine proteases important for blood digestion (Sojka et al., 2008) and host invasion (Florin-Christensen and Schnittger, 2009), respectivelly. Addition of various cysteine protease inhibitors into the *B. bovis* culture resulted in parasite growth inhibition (Okubo et al., 2007). The cysteine proteases inhibitor called cystatin-2 (Hlcyst2) from *H. longicornis* (Zhou et al., 2006) was overexpressed in midgut and hemocytes after *Babesia* infection. Recombinant HlCyst2 had slight effect on *B. bovis* growth in *in vitro* assays, but its role in the tick infection has never been experimentally examined.

Three tick genes, namely TROSPA, serum amyloid A and calreticulin has been recently identified by cDNA screen as genes upregulated after the tick infection with *B. bigemina* (Antunes et al., 2012). TROSPA is a midgut receptor with unknown function, which is used by *Borrelia* spirochete as a docking protein for midgut colonization and spirochete persistence (Pal et al., 2004). Serum amyloid A is a homolog of vertebrate acute phase protein reacting to inflammation (Urieli-Shoval et al., 2000). Calreticulin is an intracellular protein with many functions, including calcium binding, protein folding and immune signaling (Wang et al., 2012). Involvement of these genes in *Babesia* infection has been confirmed by RNAi, where silencing significantly reduced *B. bigemina* numbers in *Rhipicephalus annulatus* and *R. microplus* (Antunes et al., 2012). Furthermore, RNAi silencing of subolesin (the previously mentioned ortholog of mammalian akirin) or vaccination with recombinant SUB strongly reduced acquisition of *B. bigemina* by *R. microplus* fed on an infected cattle (Merino et al., 2011).

Vitellogenin serves as a storage protein and source of amino acids during embryogenesis and its uptake is achieved by a specific vitellogenin receptor, which was identified from *H. longicornis* and shown by RNAi to be indispensable for egg development (Boldbaatar et al., 2008). Interestingly, *Babesia* DNA was not detected in eggs lays from ticks with silenced vitellogenin receptor previously fed on dogs infected with *B. gibsoni*. This suggests that impairing the vitellogenin uptake interrupt the parasite transovarial transmission.

## Conclusion—future perspectives

The overall knowledge of tick innate immunity still lags far beyond the model invertebrate organisms and arthropod disease vectors. However, the availability of *I. scapularis* genome database (Megy et al., 2012), feasibility of functional genomics based on RNAi (De La Fuente et al., 2007b) and extensive number of tissue transcriptomes obtained from a variety of tick species promise to counterbalance experimental difficulties associated with tick handling and manipulation. Furthermore, introduction of the artificial membrane feeding (Krober and Guerin, 2007) extends our possibilities how to simulate the natural infections of ticks without the need of using laboratory animal models. These favorable conditions offer almost unlimited perspectives for the advanced research of the tick immune system and its impact on pathogen transmission. Among others, we can enumerate several high-priority topics, which can significantly aid to our understanding of the tick-pathogen relationship: (1) the role of epithelial immunity and maintenance of the redox balance for the pathogen persistence in the tick midgut; (2) interactions between the pathogens and commensal microflora; (3) tick antimicrobial peptides and their regulation via the Toll, Imd and JAK-STAT signaling pathways; (4) the role of tick primordial complement system in the immune response against transmitted pathogens; (5) tick molecules involved in the pathogen acquisition, persistence or transmission as vaccine candidates; (6) the detailed description of the pathogen transmission cycles within the tick vector. Taken together, focused research in these areas can lead to the ultimate goal of efficient control of tick-borne diseases.

### Conflict of interest statement

The authors declare that the research was conducted in the absence of any commercial or financial relationships that could be construed as a potential conflict of interest.
